# AI-Generated Personalized Visualization of the Safe Place in Virtual Reality vs Traditional Safe Place Imagery: Randomized Controlled Trial

**DOI:** 10.2196/82647

**Published:** 2026-07-24

**Authors:** Franziska Griessenauer, Franziska Pfannerstill, Thomas Probst

**Affiliations:** 1 Division of Psychotherapy and Psychotherapy Research Department of Psychology University of Salzburg Salzburg, Salzburg Austria

**Keywords:** artificial intelligence, imagery exercise, relaxation, safe place, virtual reality

## Abstract

**Background:**

Relaxation techniques, such as the “safe place” imagery exercise, are simple and accessible strategies to cope with the negative effects of stress. While virtual reality (VR) has been applied in relaxation research, it is still unclear whether it enhances the relaxing effect of such exercises or is merely an alternative and similarly effective form of delivery.

**Objective:**

This study aimed to evaluate whether the use of VR enhances the relaxing effect of the safe place imagery exercise by generating an individualized virtual environment representing each participant’s personal safe place with AI. Additionally, the roles of imagery vividness, presence, and satisfaction with the AI-generated safe place were examined.

**Methods:**

This randomized controlled trial involved a single laboratory session with 4 measurement points followed by a 5-day online follow-up period involving daily self-report questionnaires. A total of 60 adults were randomly assigned to the experimental condition or the control condition. In the experimental condition, participants engaged in the safe place imagery exercise in which they imagined a place where they feel completely safe and comfortable, followed by a stress induction task. Then, participants completed a 3-minute relaxation period within an AI-generated virtual environment representing their personal safe place, which was created based on their individual descriptions. In the control condition, the same procedures were implemented; however, in the 3-minute relaxation period, participants imagined their safe place with their eyes closed. The primary outcome was self-reported relaxation, assessed with the Relaxation State Questionnaire. Secondary outcomes included imagery vividness (assessed with the Vividness of Visual Imagery Questionnaire), presence (assessed with the Igroup Presence Questionnaire), satisfaction with the AI-generated safe place, and use of the safe place in everyday life.

**Results:**

The 3-minute relaxation period of both conditions enhanced self-reported relaxation levels with no statistically significant differences between conditions, indicating that relaxation in VR did not outperform traditional mental imagery. Relaxation levels during the 5-day online follow-up period likewise did not differ significantly between conditions, and there were no significant differences in the frequency with which participants used their safe place during the follow-up period. Imagery vividness was positively associated with postintervention relaxation in both conditions, whereas satisfaction with the AI-generated safe place and presence in the virtual environment did not predict outcomes.

**Conclusions:**

While relaxation in the AI-generated safe place in VR was effective in promoting relaxation, it did not demonstrate a benefit compared to traditional mental imagery, either immediately after the intervention or during the follow-up period. These findings suggest that VR may represent an effective alternative form of delivery, but not a superior enhancement, for brief relaxation interventions. Future developments might increase the potential of AI and VR as effective tool for promoting relaxation.

**Trial Registration:**

Open Science Framework 10.17605/OSF.IO/BP53A; https://osf.io/bp53a/overview

## Introduction

### Overview

While stress is in many cases an adaptive and natural human response that prompts us to address challenges [1], prolonged exposure to stressors over a lifetime can lead to various physiological and psychological health issues, including cardiovascular disease, chronic pain, mental illness, and certain types of cancer [2-4].

Relaxation techniques such as yoga, meditation, guided imagery, or progressive muscle relaxation are cost-effective, safe, and practical ways to manage stress [5]. An example of a guided imagery exercise is the safe place imagery exercise. This is an exercise in which a person imagines a place, real or made-up, where they feel completely safe and comfortable. The safe place imagery exercise is used in a variety of therapeutic settings and has been shown to be effective in regulating emotions and reducing negative affect, especially after exposure to trauma [6-9].

### Relaxation in Virtual Reality

Practicing such techniques in everyday life can be challenging. Modern life is filled with constant distraction and sensory stimulation from phones and other media, which can make it difficult to maintain focus and fully engage in relaxation exercises [10]. Virtual reality (VR) may offer a solution by creating immersive, controlled environments that minimize external distractions and support the practice of techniques such as the safe place imagery exercise. Due to such distractions, people practicing relaxation exercises and mindfulness often report difficulties sitting still, focusing on the exercise, and being fully present in the moment [11]. VR technology can help address this issue. VR headsets are designed to capture attention, using head-tracking technology to alter the view as the user moves their head. This creates an immersive experience, which enhances the sense of presence in the virtual environment [11]. Presence is a key factor in reducing distractibility and is defined as the subjective experience of “being there” in a virtual environment, despite physically being elsewhere [12,13]. Busy schedules also represent a common barrier to the consistent practice of relaxation exercises, as time constraints and competing responsibilities often limit individuals’ ability to engage regularly [10]. VR may potentially help with this challenge by increasing motivation and engagement through immersive and interactive experiences. Evidence from longitudinal intervention studies indicates that the use of VR can enhance adherence to mindfulness and relaxation practices, indicating its potential to support long-term behavior change even under the time pressure of daily life [14,15]. Another advantage of relaxation in VR is its ability to provide access to visual experiences such as natural environments that might otherwise be unattainable due to physical or geographical constraints [16], since, for example, access to nature remains limited for many people living in urban areas [17]. Research has shown that exposure to natural environments can enhance the effects of relaxation exercises, both in real life and simulated in VR [18-21]. Moreover, VR can potentially account for limitations in imagery capabilities, since it externalizes imagery and provides rich multisensory cues instead of relying on internal visualization [22]. However, to date, there is little empirical evidence examining how individual differences in imagery ability influence the effects of VR-based relaxation interventions.

VR relaxation interventions show promising results for stress management and relaxation, demonstrating effectiveness both in healthy adults [10] and in those with mental health conditions [23]. But evidence on whether VR can improve the effect of traditional relaxation exercises or is merely an alternative and similarly effective form of delivery remains mixed. For example, in a study in which participants took part in 2 sessions of guided meditations, one in VR and one either on a 2D screen or face-to-face with an experimenter, VR sessions were associated with a heightened experience of awe, but no difference in any other affective state compared to non-VR sessions [24]. In a study by Tarrant et al [25], participants listened to the same 5-minute–long audio of a guided meditation either with a VR headset and neurofeedback or with neither a VR headset nor neurofeedback. Both groups showed a significant decrease in anger, tension, and depression, while the VR and neurofeedback group experienced improvements in calmness and happiness and decreased vigor, fatigue, and confusion, which were not observed in the audio-only group. However, given the use of both VR and neurofeedback in the experimental condition, it remains unclear to what extent the observed effects can be attributed specifically to the VR component. In another study by Liszio et al [26], participants were exposed to either a natural environment in VR or on a 2D screen but did not receive a guided relaxation exercise. Results showed that exposure to a natural environment in VR led to a greater reduction of physiological stress, anxiety, and heightened positive affect compared to exposure to the same environment on a 2D screen, but negative affect and cortisol did not differ between the conditions [26].

Multiple studies have found no advantages of VR interventions over non-VR alternatives. For instance, a study by Poetar et al [27] revealed that mindfulness interventions delivered with and without VR resulted in comparable reductions in negative affect. In this study, participants in both conditions engaged in a session of guided breathing meditation during which the VR group floated through a surreal virtual environment and the control group looked at a 2D image of a meditating woman on a computer screen. However, comparability of the 2 groups is limited since the VR exposure included gamification, different exercises, and visuals to the control group. Similarly, no significant differences were found in the effectiveness of a long-term mindfulness intervention, regardless of whether they were supported by VR technology. For example, in a study by Malbos et al [15], participants with generalized anxiety disorder received 6 sessions of a relaxation intervention, either combined with VR or as the standard mental imagery intervention, and no difference between these groups was found in terms of the effects of the intervention. Another longitudinal study by Modrego-Alarcón et al [14], in which participants underwent a 6-week mindfulness program with or without VR support, did not show differences in stress reduction or relaxation between groups.

### Personalized Virtual Environments

An important consideration in the use of VR headsets for relaxation is the design of the virtual environment, specifically, what it should look like and how it should be created to best support the intended outcomes. New technology offers the opportunity to alter the environments that are presented to the specific needs and preferences of a person, which can be described as personalization [28]. Fan and Poole [29] describe personalization as tailoring a system to user characteristics or preferences, drawing on Blom’s [30] definition of modifying system features or content to increase personal relevance. In VR, such personalization typically involves adapting environments based on user input and has been associated with higher engagement, presence, and immersion [31-33]. Following this user-centered perspective, our method-generating safe place environments from participant keywords-constitutes a step toward personalized VR, even though the resulting scenes were static and nonadaptive. This kind of user-centered approach can be implemented through various methods. For example, Pardini et al [31] personalized elements of the virtual environment by allowing participants to choose aspects such as the setting (eg, mountains or beach), weather conditions, and ambient sounds. Their participants relaxed in both a standard and a personalized virtual environment, and most of them preferred the personalized environment. Participants also experienced higher relaxation levels and more pleasant emotions in the personalized environment. Pizzoli et al [28] suggest incorporating autobiographical memories into virtual environments to promote emotional engagement. For instance, integrating a personally meaningful place could enhance feelings of peace and security [34].

### Present Study

This study tested fully personalized virtual environments of the imagined safe place generated by AI (experimental condition: relaxation in a personalized virtual safe place [IMG+VR]) against the imagination of the safe place without personalized virtual environments generated by AI (control condition: relaxation while imagining a safe place with closed eyes [IMG]). The personalization in our study goes beyond selecting from a set of predefined VR environments, such as those used by Pardini et al [31] and Pardini [32]. Instead, each virtual safe place is generated solely on the basis of the participant’s own individually provided keywords, resulting in a unique environment that reflects their personal associations and preferences. Although the environment is not interactively adaptable in real time, this generative approach represents a relevant next step in personalization because it creates individualized content rather than offering a limited set of standard options. To our knowledge, this is the first study to examine the use of virtual environments that were constructed with AI in the context of relaxation. The following hypotheses were tested:

- H1a: participants of the IMG+VR condition experience higher levels of relaxation compared to participants of the IMG condition after the intervention during the laboratory session.
- H1b: participants of the IMG+VR condition experience higher levels of relaxation compared to participants of the IMG condition in the 5-day follow-up period.
- H2 (exploratory): imagery vividness moderates the effect of the intervention on relaxation.
- H3a: higher satisfaction with the AI-generated safe place leads to higher relaxation levels after the intervention in the IMG+VR condition.
- H3b: higher presence scores lead to higher relaxation levels after the intervention in the IMG+VR condition.
- H4: the visual representation of the safe place in the IMG+VR condition leads to an increased use of the safe place in everyday life compared to the IMG condition.

Moreover, for exploratory purposes, the participants’ satisfaction with their personal safe place was evaluated. 

## Methods

### Study Design

This study used a between-subjects experimental design with repeated measures to investigate the effects of integrating VR and AI into a single session of the safe place imagery exercise on participants’ relaxation levels. Outcomes were measured with questionnaires through ESMira [35], a mobile app designed for conducting experience sampling method studies. ESMira is installed on an internal server of the Paris Lodron University of Salzburg, and the collected data are stored on the same server.

The randomized controlled trial compared 2 conditions. An experimental condition in which participants received an AI-generated virtual environment representing their individual safe place (IMG+VR) and a control condition, where participants imagined their safe place without the use of a VR headset (IMG). Randomization was carried out using the website Randomizer [36], which generated a random list of numbers from 1 to 60. The IMG+VR condition was then assigned to the even numbers and the IMG condition to the odd numbers. The analysis plan of the data was preregistered at the Open Science Framework on February 8, 2025.

### Participants and Recruitment

The target sample size was 60 participants. This number was based on feasibility and sample sizes used in previous studies with comparable designs and interventions, rather than on a formal a priori power analysis. Participants were recruited through interpersonal contact or advertisements on the study registration platform SONA [37]. Any German-speaking person aged 18 years or older with a smartphone to install the app ESMira was eligible to participate.

### Ethical Considerations

This study involved human participants and was reviewed and approved by the Ethics Committee of the University of Salzburg (registration number GZ 43/2024). All participants provided written informed consent prior to participation. The consent procedure included detailed information about the study procedures, potential risks, voluntary participation, and the right to withdraw at any time without negative consequences. Participant privacy and confidentiality were strictly protected. All data were collected in pseudonymized form; digital data were stored on servers of the University of Salzburg; physical data were kept locked in the premises of the Outpatient Clinic for Psychotherapy of the Paris Lodron University of Salzburg. Data were anonymized for data analyses. Only aggregated results are reported.

Participating psychology students received 2 hours of credit; all other participants received no compensation. Exemplary screenshots of the VR environments in Multimedia Appendix 1 are included with the explicit consent of the respective participants for publication purposes.

### Procedure

Every participant completed one in-person laboratory session, followed by 5 daily online follow-up assessments.

### Laboratory Session

#### Overview

All laboratory sessions took place in the premises of the Outpatient Clinic for Psychotherapy of the Paris Lodron University of Salzburg and lasted approximately 1 hour. All sessions were conducted by the first author. Each session began with the distribution of the study information, signing of the consent form and installation of the ESMira app on the participant’s smartphone. Then a baseline questionnaire was completed. Afterward, the safe place imagery exercise was introduced. The experimenter left the room while the participants listened to a 12-minute audio recording of the safe place imagery exercise by Reddemann [38]. The participants then completed a short questionnaire (Postimagery questionnaire) followed by a worksheet in which they described their safe place in 3-5 keywords. To induce nonrelaxed, stressed states in all participants, they were then required to undertake a cognitive test with time pressure consisting of very challenging matrix reasoning tasks based on Raven’s Progressive Matrices on a tablet provided by the experimenter. The task took about 5 minutes and is a part of the Salzburg Mobile Stress Induction [39], which was specifically developed to reliably induce nonrelaxed, stressed states. During the laboratory session, participants were unaware of the true purpose of the task; however, at the end of the laboratory session, they were informed that completing the task successfully within the time limit was not possible. After the stress induction task, participants filled out another short questionnaire (Poststress Questionnaire). From then on, the procedures of the two conditions differed.

#### IMG+VR Condition

A Meta-Quest 3 VR headset with the application PsyTechVR [40] was used for the generation of the individualized safe place. The experimenter typed the keywords the participant had previously written down on the worksheet into the application, and an AI built into the application called MindGap AI then generated a photorealistic 360-degree virtual environment based on the keywords. The created environment was a static visual 3D environment without movements of the participant/objects and without sounds. The custom AI system was based on a latent diffusion model (a text-to-image architecture comparable to Stable Diffusion) capable of generating 360° panoramic scenes from textual descriptions or simple sketches [41]. For spatially richer outputs, the system used a depth-aware extension from the Latent Diffusion Model for 3D (LDM3D) family. We used the 2024-2025 version of this model, which was trained and fine-tuned on a large dataset of paired image-depth-caption samples (red-green-blue image, depth map, and text caption). The optional AI-based prompt enhancement was not used, and no prompt engineering or automatic augmentation was applied. The same set of keywords could yield different environments on different generations due to the stochastic nature of the diffusion model. No adaptations were possible, as generating a single environment required approximately 30-60 seconds. Additional delays were avoided to prevent potential effects on participants’ relaxation levels. See Multimedia Appendix 1 for exemplary environments.

Participants were asked to put on the headset, immerse themselves in the environment for 3 minutes, and relax. They were not allowed to modify their virtual safe place. Afterward, the experimenter took a screenshot of the participant’s safe place in the VR headset and included that picture in the follow-up questionnaires for that participant.

#### IMG Condition

Participants in the IMG condition were asked to close their eyes, imagine their personal safe place, and relax for 3 minutes.

At the end of the laboratory session, participants of both conditions completed a questionnaire (Postintervention questionnaire).

### Follow-Up Period

The follow-up questionnaires were distributed daily at 6 PM via the ESMira app for 5 days after the laboratory session. The questionnaires included questions about whether they thought about their safe place that day and whether a challenging situation prompted them to do so. Participants in the IMG+VR condition were shown a screenshot of their AI-generated safe place and instructed to mentally revisit it for a moment. Participants in the IMG condition were asked to recall their safe place and briefly imagine themselves being in it. The follow-up questionnaires were available for 24 hours on days 1 to 4, starting 24 hours after the laboratory session, whereas the day 5 questionnaire remained open indefinitely, thereby allowing for late submissions. A push notification was sent at 6 PM to remind participants to complete the questionnaire, and participants had the option to modify this reminder time individually.

### Outcome Measures

All outcome measures were self-reported through questionnaires.

### Primary Outcome Measure—Relaxation

The Relaxation State Questionnaire (RSQ) [42], the primary outcome of this study, measures current physical and psychological relaxation with 10 items such as “My muscles feel relaxed.” rated on a Likert scale from 1 (“not correct at all”) to 5 (“entirely correct”), with higher values indicating higher relaxation. It shows good internal consistency (Cronbach α=0.86) [42]. The RSQ was assessed at baseline, postimagery, poststress, postintervention, and follow-up.

### Secondary Outcome Measures

#### Imagery Vividness

The Vividness of Visual Imagery Questionnaire (VVIQ) [43] measures the clarity and vividness of visual mental imagery using 16 items. At baseline, participants were asked to visualize specific scenarios or objects and then rate how vivid or clear these mental images were. One example item is: “Visualize a rising sun. Consider carefully the picture that comes before your mind's eye. Then rate the following items. The sun is rising above the horizon into a hazy sky.” It is scored on a Likert scale ranging from 1 (“No image at all”) to 5 (“Perfectly clear and as vivid as normal vision”), with higher values indicating higher imagery vividness. It shows high internal consistency (Cronbach α=0.91) and high construct validity [44].

#### Presence

Presence is a psychological construct that describes the subjective sense of being in a virtual environment [12,13]. The Igroup Presence Questionnaire (IPQ) [45] is a tool to measure this construct with 14 items such as “I was completely captivated by the virtual world” on a 7-point Likert scale ranging from –3 to +3, with higher scores indicating higher presence. It shows acceptable to good internal consistency on all subscales: spatial presence (Cronbach α=0.78), involvement (Cronbach α=0.74), and experienced realism (Cronbach α=0.63) [46]. The total scale of the IPQ was used for calculations in this study as suggested by Tran et al [47]. Cronbach α for the total scale was 0.85 and 0.87 in 2 preliminary studies [48]. The IPQ was administered postintervention in the IMG+VR condition immediately after participants removed the headset.

#### Satisfaction With Safe Place

At postintervention, the participants’ satisfaction with their individually generated safe place was operationalized with 4 items. The first item asked participants to rate how much the AI-generated environment matched the imagination they initially had of their safe place on a visual analog scale from 1 (“not at all”) to 100 (“completely”). The second item was a binary item: “Did you receive the picture you wanted?” The third question invited participants to rate their satisfaction with their safe place on a Likert scale ranging from 1 (“pretty unsatisfied”) to 4 (“very satisfied”). Finally, participants were invited to provide feedback regarding any changes they would make to the virtual environment if given the opportunity to do so in an open-text question.

#### Usage of the Safe Place

In the daily follow-up questionnaires, participants were asked whether they had thought of their safe place on that day and if there was a specific challenging situation in which their safe place came to their mind. If any of those questions were answered with “yes,” participants received a question asking them to rank how they felt after thinking of their safe place on a Likert scale ranging from 1 (“very tense”) to 10 (“very relaxed”).

### Data Analysis

SPSS Statistics (version 29; IBM Corp) software was used for statistical analyses. All tests were performed 2-tailed with a significance value of *P*<.05. A manipulation check was conducted to assess the effect the safe place imagery exercise and the stress induction had on the subjective relaxation levels of the participants. This was done using dependent *t* tests comparing baseline RSQ with postimagery RSQ and postimagery RSQ with poststress RSQ in both conditions separately. Baseline group equivalence was assessed using independent-sample *t* tests, Mann-Whitney *U* tests, chi-square tests, or Fisher exact tests, depending on the distribution and scale of the variables. Primary and exploratory hypotheses were prespecified. No formal correction for multiple comparisons was applied.

To test the immediate effect of the intervention in the IMG+VR condition compared to the IMG condition (H1a), a mixed ANOVA was conducted with relaxation levels before (poststress RSQ) and after (postintervention RSQ) the intervention as the within-subject factors and condition as the between-subject factor.

To analyze whether relaxation levels differed between conditions in the follow-up period, a linear mixed model was used. This approach was chosen, although it differed from the preregistered analysis plan, because the extent of missing data at follow-up was greater than anticipated. The linear mixed effects model allowed us to retain participants with incomplete data and to account for unequal numbers of observations per participant. The model included condition (IMG vs IMG+VR) as a fixed effect, with a random intercept for participants to account for repeated measurements. The model was estimated using maximum likelihood, and fixed effects were evaluated using Satterthwaite-adjusted degrees of freedom. Follow-up time points were not included as a fixed effect, as model comparisons showed that models containing time did not improve fit and our hypotheses focused on overall condition differences rather than time‑specific effects.

To examine whether the main findings of H1 were influenced by participants’ satisfaction with the intervention, sensitivity analyses were conducted excluding participants who indicated that they were not satisfied with the VR environment they received.

A moderation analysis was conducted in an exploratory manner with the *PROCESS* macro for SPSS (version 4.2) to examine whether imagery vividness influences the effect of the condition (IMG+VR vs IMG) on relaxation at postintervention, while controlling for relaxation after stress induction (H2).

To assess the role of satisfaction with the AI-generated safe place (H3a) and presence (H3b), 2 regression analyses were calculated with the RSQ score of the IMG+VR participants at postintervention as the dependent variable and the relaxation levels before the intervention (poststress RSQ) as well as satisfaction with the AI-generated safe place or presence as predictors.

To compare the daily use of the safe place between the two conditions, an independent *t* test with Welch’s correction was used to account for unequal variances. To account for missing data in the follow-up period, the dependent variable was the number of days in which participants reported thinking of their safe place (assessed by the following item: “Have you thought about your safe place today?” [yes/no]) in relation to the number of days they completed the follow-up questionnaires. As a robustness check for the bounded and variable proportional data, a nonparametric Mann-Whitney *U* test was additionally conducted.

The responses of participants concerning their satisfaction with the AI-generated safe place were analyzed in an explorative manner using descriptive statistics. Open-text responses regarding desired changes to the AI-generated environments were reviewed and grouped into inductively derived thematic categories for exploratory summarization.

## Results

### Sample Description

A total of 60 people participated in this study. For sociodemographic characteristics, see Table 1. Participants in the IMG+VR condition were, on average, 33.67 (SD 17.48) years of age and had a median age of 25.00 (IQR 20.75-43.75) years, indicating a right-skewed distribution. Participants of the IMG condition were, on average, 27.70 (SD 11.79) years of age and had a median age of 23.00 (IQR 21.00-29.50) years, suggesting a less pronounced skew. Participants’ age did not differ between conditions as assessed with the Mann-Whitney *U* test (*U*=389.00; *Z*=–0.90; *P*=.37). In the RSQ at baseline, participants of the IMG condition had a mean score of 3.21 (SD 0.65), participants of the IMG+VR condition had a comparable mean score of 3.07 (SD 0.56; t_58_=–0.85; *P*=.40). In the VVIQ at baseline, participants of the IMG condition had a mean score of 63.2 (SD 11.2) and participants of the IMG+VR condition had a mean score of 64.0 (SD 10.7) with no differences between the conditions (t_58_=0.27; *P*=.79). Correlations among key variables are reported in Table S1 in Multimedia Appendix 2.

**Table 1:** Sociodemographic characteristics of participants and results of the baseline group comparison tests.

Baseline characteristics		IMG+VR^a^, n (%)	IMG^b^, n (%)	Test statistics		Effect size		*P* value	
**Gender**					N/A^c^		0.07		>.99
	Male	6 (20)	7 (23)						
	Female	21 (70)	21 (70)						
	Other	3 (10)	2 (7)						
**Highest educational level**					N/A^c^		0.15		.61
	Middle school	1 (3)	0 (0)						
	High school	27 (90)	29 (97)						
	Other	2 (7)	1 (3)						
**Employment**					N/A^c^		0.25		.17
	Studying	19 (63)	25 (84)						
	Employed	6 (20)	4 (13)						
	Retired	5 (17)	1 (3)						
Previous experience with virtual reality		11 (37)	15 (50)	1.09 (1)^d^		0.14		.30	
Previous experience with relaxation methods		25 (83)	25 (83)	0.00 (1)^d^		0.00		>.99	

^a^IMG+VR: experimental condition (relaxation in a personalized virtual safe place).

^b^IMG: control condition (relaxation while imagining a safe place with closed eyes).

^c^Fisher exact test.

^d^Chi-square test (*df*).

**Figure 1:**
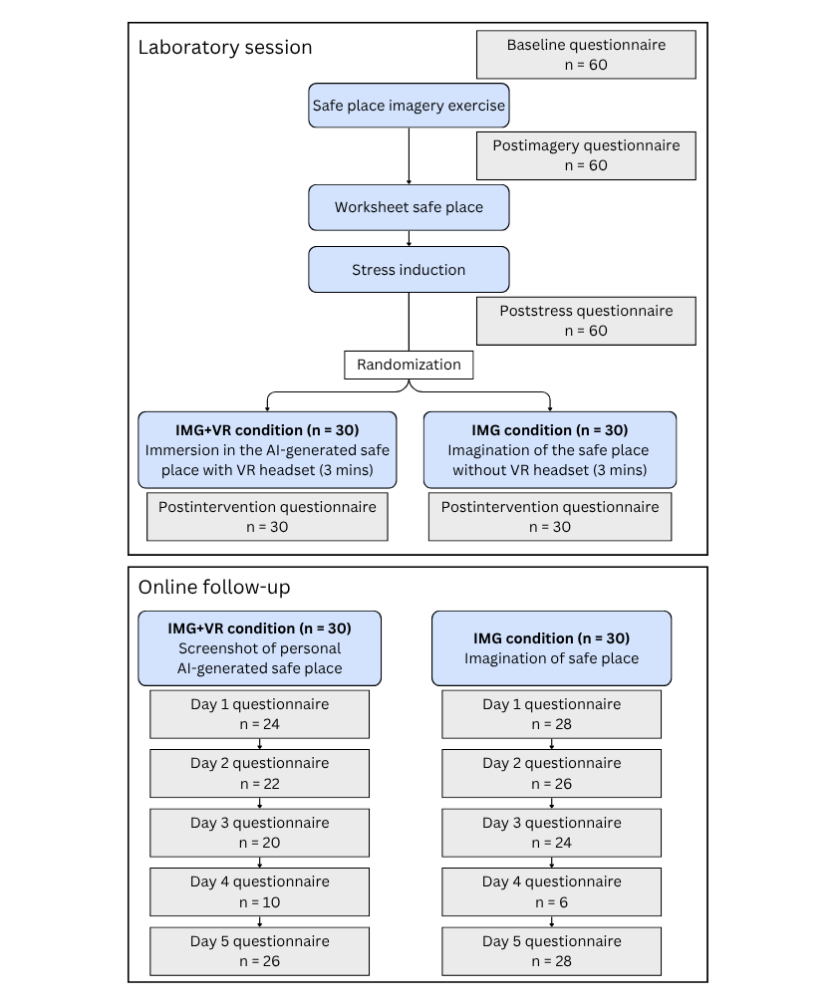
Experimental procedure and study flow of the randomized controlled trial (N=60 adults). After enrollment and random assignment to either the experimental condition (relaxation in a personalized virtual safe place [IMG+VR]) or the control condition (relaxation while imagining a safe place with closed eyes [IMG]), participants completed a laboratory session of around 1 hour in the premises of the Outpatient Clinic for Psychotherapy of the Paris Lodron University of Salzburg. Participants engaged in 4 tasks during the laboratory sessions: a safe place imagery exercise (12 minutes), filling out a worksheet describing their safe place in 3-5 words, a stress induction task (5 minutes), and a 3-minute relaxation intervention. In the IMG+VR condition, relaxation was conducted in an AI-generated, personalized virtual environment representing the participant’s safe place; in the IMG condition, relaxation was performed using mental imagery with closed eyes. The laboratory session included 4 measurement points: the baseline questionnaire (Relaxation State Questionnaire [RSQ], Vividness of Visual Imagery Questionnaire), the postimagery questionnaire (RSQ), the poststress questionnaire (RSQ), and the postintervention questionnaire (RSQ, Igroup Presence Questionnaire, satisfaction with the AI-generated safe place). All questionnaires in the laboratory session were filled out on a tablet provided to the participants. The laboratory session was followed by a 5-day online follow-up period during which participants completed daily self-report questionnaires assessing relaxation (RSQ) and use of their safe place each day.

Participants completed an average of 71% (SD 24%) of the 5 daily follow-up questionnaires. Completion rates declined from day 1 to day 4 and increased again on day 5 (for exact numbers, see Figure 1). Little missing completely at random test was nonsignificant (*χ*^2^_32_=31.15; *P*=.51), suggesting no statistical evidence that missingness was related to observed variables.

### Descriptive Statistics of Relaxation Scores at the Assessment Points of the Study

**Figure 2:**
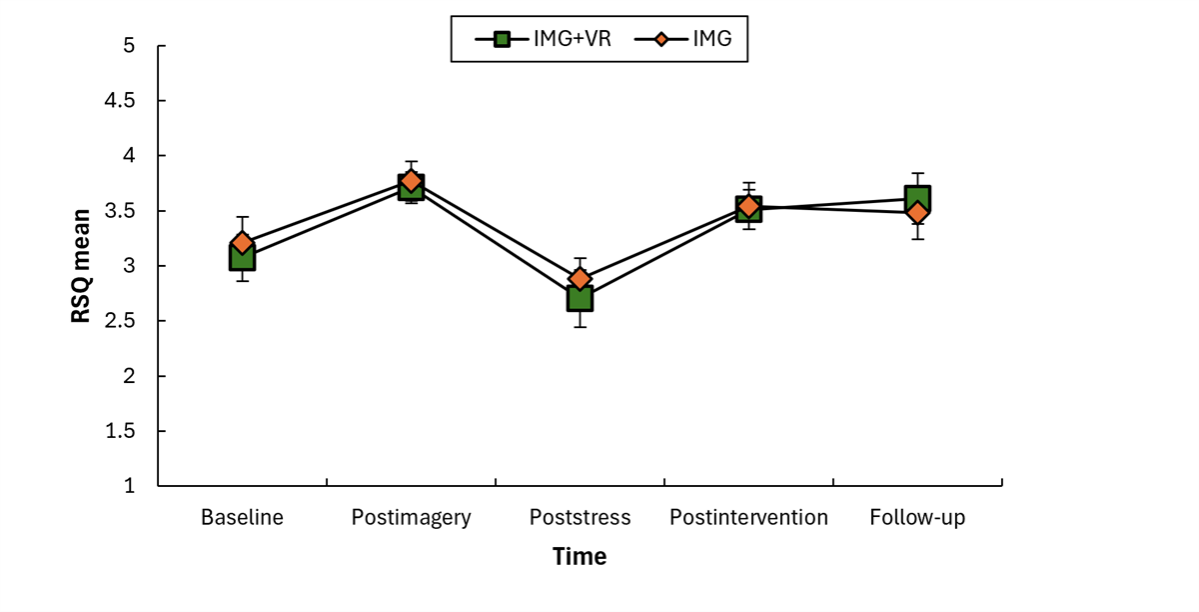
Trajectory of self-reported relaxation scores. Relaxation levels in both conditions during the randomized controlled trial. Error bars represent SEs of the mean. Measurements were obtained at baseline, after a guided safe place imagery exercise (postimagery), following a stress induction task (poststress), and after a 3-minute relaxation intervention (postintervention). The follow-up data point represents the aggregated mean of all available relaxation scores across the 5-day follow-up period and is shown for visual simplicity. Statistical analyses were conducted using all available daily follow-up measurements within a linear mixed effects model. IMG: control condition (relaxation while imagining a safe place with closed eyes); IMG+VR: experimental condition (relaxation in a personalized virtual safe place); RSQ: Relaxation State Questionnaire.

Figure 2 shows trajectories of relaxation scores on the outcome RSQ during the study. For detailed descriptive statistics, including tests for normality, see Table S2 in Multimedia Appendix 3.

### Manipulation Check

The safe place imagery exercise showed a statistically significant increase (comparison baseline RSQ vs postimagery RSQ) while the cognitive task induced a statistically significant decrease (comparison postimagery RSQ vs poststress RSQ) in subjective relaxation levels (RSQ) in both conditions, as indicated by pairwise *t* tests (all *P*<.001; Table S3 in Multimedia Appendix 4).

### Results for Hypothesis 1a

A mixed ANOVA was conducted to compare relaxation levels before the intervention (poststress RSQ) to after (postintervention RSQ) between the two conditions. The assumption of normality in the RSQ scores was tested separately for each condition and time point using the Shapiro-Wilk test (Multimedia Appendix 3). At postimagery, the test was significant in the IMG+VR condition, indicating a deviation from normality (*P*=.045), as well as at postintervention in the IMG+VR condition (*P*=.01). However, for all other points of measurement in both conditions, the tests were nonsignificant (*P*>.05), suggesting normal distribution of the data. Given that the mixed ANOVA is relatively robust against moderate violations of normality, we proceeded with the analyses [49]. There was no statistically significant interaction between time of measurement and condition (*F*_1, 58_=0.83; *P*=.37; partial η^2^=0.014, 95% CI 0.00-0.12). There was a significant main effect for time of measurement (*F*_1, 58_=87.0; *P*<.001; partial η^2^=0.600, 95% CI 0.43-0.70) and no significant main effect for the condition (*F*_1, 58_=0.72; *P*=.40; partial η^2^=0.012, 95% CI 0.00-0.11). Therefore, the intervention significantly increased relaxation levels regardless of whether or not a VR headset was used.

### Results for Hypothesis 1b

A linear mixed model was conducted to compare relaxation levels between conditions in the follow-up period. There was no significant main effect of condition (*F*_1, 55.36_=3.16; *P*=.08), suggesting that relaxation levels did not differ between conditions across the follow-up period.

Excluding participants with low satisfaction did not meaningfully alter the pattern of results for H1a or H1b. The interpretation of main effects and interactions did not change (Multimedia Appendix 5).

### Results for Hypothesis 2

A moderation analysis was conducted in an exploratory manner to assess if imagery vividness, assessed with the VVIQ, influences the effect of the condition on relaxation after the intervention (postintervention RSQ) while controlling for poststress relaxation. The overall model of the moderation analysis was significant (*F*_4, 55_=8.19; *P*<.001; *R*^2^=0.37). Imagery vividness did not moderate the effect of condition on relaxation (Δ*R*^2^<0.01%; *F*_1, 55_=0.03; *P*=.85). The interaction term was not significant (*B*=0.00, SE 0.01, 95% CI –0.02 to 0.02).

For interpretational purposes, effects were examined within the full model. As suggested by Hayes [50], the interaction was not further probed given its nonsignificance. The analysis showed a significant main effect of imagery vividness (*B*=0.02, SE 0.01, 95% CI 0.01-0.04; t_55_=2.66; *P=*.01), suggesting that higher imagery vividness is associated with greater relaxation levels after the intervention regardless of the condition. Poststress relaxation also significantly predicted postintervention relaxation (*B*=0.31, SE 0.09, 95% CI 0.12-0.50; t_55_=3.28; *P*=.002). The main effect of condition was not significant (*B*=–0.13, SE 0.67, 95% CI –1.465 to 1.210; t_55_=–0.19; *P*=.85).

### Results for Hypothesis 3a

A regression analysis with data from the IMG+VR condition was conducted with the dependent variable “postintervention RSQ” and the predictors “poststress RSQ” and satisfaction with the AI-generated safe place (1-100). The overall model was not statistically significant (*F*_2, 27_=1.62; *P*=.22). The *R*^2^ for the overall model was 0.11 (adjusted *R*^2^=0.04). Neither relaxation before the intervention (poststress RSQ), *B*=0.23, SE 0.13; *β*=.33, 95% CI –0.03 to 0.49; t_27_=1.80; *P*=.08, nor satisfaction with the safe place, *B=*0.00, SE 0.00; *β*=.10, 95% CI –0.01 to 0.01; t_27_=0.55; *P*=.59, predicted relaxation after the intervention in the IMG+VR condition.

### Results for Hypothesis 3b

Another regression analysis with data from the IMG+VR condition was conducted with the dependent variable postintervention RSQ and the predictors poststress RSQ and presence (IPQ). Participants in the IMG+VR condition reported a mean presence score of 0.76 (SD 0.79) on the IPQ. The overall model of the regression analysis was not statistically significant (*F*_2, 27_=2.17; *P*=.13; *R*^2^=0.14; adjusted *R*^2^=0.08). Both relaxation before the intervention (poststress RSQ; *B*=0.22, SE 0.12; *β*=.32, 95% CI –0.03 to 0.47; t_27_=1.81; *P*=.08) and sense of presence (IPQ; *B*=0.12, SE 0.11; *β*=.20, 95% CI –0.10 to 0.34; t_27_=1.14; *P*=.27) did not predict relaxation after the intervention (postintervention RSQ) in the IMG+VR condition.

### Results for Hypothesis 4

There were 2 participants in each condition who did not fill out any questionnaires in the follow-up period, which left 56 participants for this analysis. Participants in the IMG condition thought of their safe place on average on 44.1% (SD 20.0%) of the days in which they filled out the questionnaire, and participants in the IMG+VR condition on 35.8% (SD 33.8%) of days. There was no statistically significant difference in the daily usage of the safe place between the 2 conditions (t_43.84_=–1.12; *P*=.27; *d*=–0.30, 95% CI –0.83 to 0.23). A Mann-Whitney *U* test was conducted as a robustness check, which likewise indicated no statistically significant difference between conditions (*U*=304.00; *Z*=–1.46; *P*=.14).

### Exploratory Analysis: Satisfaction With the AI-Generated Safe Place

This is the first study, to our knowledge, to use AI to create a fully personalized safe place in VR for each participant. Therefore, it is of particular interest to examine how satisfied participants were with their AI-generated virtual environment.

When asked whether participants received the environment they wanted, 23/30 (77%) participants agreed, while 7 (23%) said they did not. When asked to rate their satisfaction with the environment on a scale from 1 to 100, the answers were normally distributed around a mean of 66.00 (SD 21.14), ranging from 21 to 95. In an open-text question, participants were asked to state elements they would change about the virtual environment. A total of 5 participants indicated they would have changed the look of certain objects in the environment, and 5 would have added or removed certain objects. Overall, 11 participants would have changed the look of the landscape in which they were, and 3 would have preferred to be in a different position within the environment. One participant would have liked to add movement to the environment, another participant sounds.

## Discussion

### Principal Findings

The aim of this study was to investigate whether relaxing in an AI-generated virtual environment representing an individual’s safe place enhances subjective relaxation levels compared to a traditional imagery-based exercise, within a single 3-minute session in adults. Both the experimental condition (IMG+VR; relaxation in a personalized virtual safe place) and the control condition (IMG; relaxation while imagining a safe place with closed eyes) resulted in comparable improvements in relaxation levels after the 3-minute intervention period. Relaxation levels did not differ between conditions across the 5-day follow-up period. While the effect of the condition on relaxation was not moderated by imagery vividness, it was demonstrated that higher levels of imagery vividness resulted in higher levels of relaxation in both conditions. In the IMG+VR condition, neither the satisfaction with the AI-generated safe place nor the sense of presence influenced the relaxation levels after the intervention. The participants in the IMG+VR condition did not think of their safe place more often than participants in the IMG condition during the follow-up period.

The improvement of relaxation after the VR intervention in this study is in line with the currently existing research body. Reviews show that relaxing in VR can be beneficial for relaxation, mindfulness, regulating emotions, and experiencing positive emotions [10,23,51]. Contrary to our hypotheses, the IMG+VR condition did not improve relaxation more effectively than the IMG condition. Similar results were found where both a VR condition and an active control condition led to the same amount of reduction in negative emotions [27], negative affect, and heart rate as an indication for relaxation [52] or anger, tension, and depression [25] after a single session. Superiority of VR interventions compared to non-VR interventions was observed only in combination with neurofeedback [25] or in certain but not all affective states [24,26]. In light of the current findings and existing literature, it appears reasonable to conclude that practicing relaxation exercises can effectively increase relaxation, but VR may not provide additional benefits regarding subjective relaxation after a single session.

It was examined in an explorative manner whether imagery vividness moderates the effect of the intervention on relaxation. Specifically, individuals with lower imagery ability were expected to relax more with the visual representation of their safe place in VR than individuals with higher imagery ability. The present data did not provide evidence for this effect. One possible explanation for this absence of a moderation effect is the limited variability in imagery ability within the sample. On average, participants demonstrated high imagery ability, with only 1 individual scoring below 32 out of a maximum of 80 on the VVIQ [43], a threshold indicative of impaired visual imagery [53]. This inability to generate mental images is known as aphantasia [54] and affects only around 3.9% of the general population [53]. The lack of scores at the lower end in the VVIQ limits the interpretability of the moderation analysis and reduces the statistical power to detect potential differences in how the intervention affects relaxation across a wider variety of imagery ability. Future research should aim to examine the effects of VR-supported imagery exercises specifically in individuals with aphantasia, to evaluate whether VR can compensate for reduced or absent imagery ability and thereby enhance intervention effectiveness in this population. The present results confirmed that higher imagery vividness led to greater relaxation in both conditions, replicating earlier findings that individuals with higher imagery ability report stronger subjective benefits from relaxation [55]. Another possibility for the lack of a moderation effect is that moderation analyses typically require larger sample sizes than main-effect analyses, and this study may have been underpowered to detect small interaction effects. Thus, the observed Δ*R*^2^ of 0.00% may reflect a type II error rather than the absence of a true moderating effect.

Contrary to our predictions, participants’ sense of presence in the virtual environment had no influence on their relaxation levels after the intervention. Presence is commonly considered a crucial element in eliciting authentic emotional responses within virtual environments [56,57]. This has been particularly well-documented in the field of VR exposure therapy, where higher presence has been linked to higher fear [57] and anxiety [58]. However, this relationship may not generalize to all emotional states. Freeman et al [59] proposed a theory claiming that presence is primarily associated with arousing stimuli that evoke, for example, fear, since such experiences require more alertness and attention to the environment, which corresponds to a heightened sense of presence in a virtual environment. From this perspective, presence may play a less central role in low-arousal experiences that are characterized by reduced environmental monitoring and attentional demands. Less arousing emotional states, such as relaxation, therefore correlate less with presence [60]. This was observed in a study by Standen et al [61] where higher presence was linked to higher experience of fear in participants who were exposed to fear-evoking stimuli, but not higher relaxation in participants exposed to relaxing stimuli. Although this theory still needs rigorous testing, it offers a plausible explanation as to why presence did not influence relaxation outcomes in the present data.

Participants in the IMG+VR condition did not report thinking about their safe place more frequently during the follow-up period compared to those in the IMG condition. Importantly, the study included daily ecological follow-up assessments to capture the use of the safe place exercises in everyday life. Although follow-up data were incomplete, these assessments provide preliminary insight into real-world transfer beyond the laboratory context. 

### Limitations

As the study is a randomized controlled trial, it has high internal validity but limited external validity. The sample consisted primarily of psychology students at a single university, who may be more familiar with relaxation techniques and more sensitive to experimental demands than the general public. This may have led to an overestimation of the observed effects compared to a more naïve population. The high effectiveness of the intervention in the present sample may also suggest a potential ceiling effect, which could have reduced the likelihood of detecting additional benefits of VR over imagery alone.

Given the sample size of 60 (30 per group) and α=.05, the study had 80% power to detect only large between-group effects (Cohen *d*≈0.74). Smaller effects may exist but were not detectable in this study. Nevertheless, the within-group improvements in relaxation were statistically significant, demonstrating that both VR and imagery interventions can effectively enhance relaxation in the short term.

Participants were assigned to conditions using a randomized sequence of the numbers 1-60 (even numbers to IMG+VR, odd numbers to IMG). Participants were assigned in the order in which they took part, meaning that the first participant received the condition corresponding to the first number in the randomized sequence, the second participant the condition corresponding to the second number, and so on. The allocation sequence was generated and implemented by the experimenter, who was therefore aware of condition assignment during the intervention. We acknowledge that the absence of experimenter blinding may represent a potential source of bias, although steps were taken to standardize intervention administration and instructions across participants.

The AI-generated safe place in VR consisted of a static 3D environment that comprised neither movements of the participant/objects within the safe place nor sounds. This design choice was primarily driven by the technical characteristics of the available VR platform (PsyTechVR), which supported the generation of static, silent environments based on textual input and reflects the current stage of development of psychotherapeutic VR applications. As a result, the present implementation could potentially lead to an underestimation of VR’s potential. More interactive and modifiable environments, including sounds fitted to the environment, might have more potential to enhance the effects of relaxation interventions such as the safe place. Future studies should examine more sophisticated and interactive VR environments to better capture the potential advantages of VR-based relaxation interventions.

Another limitation refers to the degree and implementation of personalization. The AI generated a static, silent image based on just 3-5 keywords that participants were not able to change or adapt. The study's own results confirm that the degree of personalization can be optimized, as 23% (7/30) of participants in the experimental group reported they did not receive the picture they wanted, and the mean satisfaction score was 66 out of 100. Future research should enhance personalization by allowing users to refine or adjust the generated environment, increasing interactivity, and incorporating motion and sound to create a more multisensory experience.

Furthermore, only a single session was conducted, which may limit the ecological validity and generalizability of the findings. It is possible that repeated or longer interventions could produce stronger or different effects, particularly in terms of sustained relaxation or cumulative benefits. Nevertheless, even this brief, single-session intervention resulted in statistically significant improvements in self-reported relaxation, suggesting that VR- and imagery-based interventions can have immediate, measurable effects.

Another limitation concerns the assessment of relaxation, which relied exclusively on a self-report questionnaire. Such measures are frequently influenced by the respondent’s personality traits such as neuroticism [62] and often correlate only moderately or not at all with objective measures [63]. Consequently, this study does not allow conclusions about autonomic nervous system regulation or potential dissociations between subjective and physiological relaxation responses. In particular, it cannot be ruled out that the VR condition elicited distinct physiological patterns despite comparable self-reported relaxation levels, as suggested by previous research showing VR-related advantages primarily on physiological but not consistently on subjective measures [26]. The inclusion of objective physiological indicators such as heart rate variability [64] could potentially provide a more comprehensive and less biased evaluation, and is suggested for future studies.

It is important to consider a methodological and a practical limitation in the measurement of the daily usage of the safe place. First, it was only measured whether a participant had thought of their safe place each day, not how often. This could lead to an underestimation of the actual amount of time participants have thought of their safe place during the follow-up period, since participants may have recalled or used the imagery multiple times per day. Second, many participants did not fill out all 5 questionnaires in the follow-up period, and the missing data reduces confidence in the observed results. Participants were scheduled to receive a single notification per questionnaire every evening and had 24 hours to complete it. Missing data on day 4 can likely be attributed to the interaction between the 24-hour activation window and the fixed notification time (PM). For participants who enrolled earlier in the day, the 4-day window ended before the scheduled notification, meaning that the final prompt was not delivered and participants were not reminded of the questionnaire for day 4. This should be considered when interpreting the reduced response rates on the fourth study day. Additionally, implementing 1 or 2 reminders could have potentially improved response rates. Furthermore, the compensation structure may have contributed to differential adherence, as psychology students received course credit regardless of their follow-up participation, whereas other participants received no compensation. This may have introduced systematic differences in response behavior between participant subgroups. Future studies should consider implementing reminder systems and more consistent incentive structures to enhance compliance.

The observed higher completion rate on day 5 likely reflects the scheduling logic of the data collection platform (ESMira). The final questionnaire was administered separately from the assessments on days 1 to 4, with its notification sent at 6 PM, 5 days after the laboratory session. In contrast to the earlier assessments, the final questionnaire remained accessible beyond the intended 24-hour window. As a result, some participants completed the assessment with a delay (mean 0.22, SD 0.54 days). Specifically, 44 participants responded within the intended time frame, 9 responded 1 day later, and 1 participant responded after 3 days, which may have contributed to the apparent recovery in response rates on day 5.

Additionally, participants in the IMG+VR condition were shown a screenshot of their AI-generated safe place during the daily follow-up assessments, whereas participants in the IMG condition received only a text-based instruction to recall their safe place. This difference in prompt format may have influenced daily engagement with the safe place imagery and should be considered when interpreting the findings related to follow-up usage.

Also, health status was not operationalized beyond general inclusion criteria, and participants may therefore not uniformly represent a healthy population. Future studies should assess these characteristics more systematically to allow for subgroup analyses and to better understand whether and how such factors moderate the effects of VR-based relaxation interventions.

### Implications

The findings of this study indicate that viewing an individual’s safe place in a virtual environment can be similarly effective in promoting relaxation as imagining it with closed eyes. Therefore, it can be regarded as a viable alternative for delivering relaxation exercises with various advantages compared to traditional relaxation exercises. These advantages include reduction of distractibility since practitioners are less aware of their in vivo surroundings and bodily discomforts during VR sessions [16].

Although this study did not investigate this directly, VR interventions may be especially helpful for individuals with lower imagery ability or higher susceptibility to distraction. Examining these effects in future research in diverse populations could inform the development of accessible, AI-supported VR interventions for digital mental health applications and real-world use.

In this study, AI was able to create environments that aligned well with many participants’ expectations even though the keywords were only entered once and no modifications to the environments were possible. While not all participants received their ideal safe place, the results highlight the large potential AI has, combined with VR, especially since in practice, users have the ability to modify environments as much as needed to meet expectations with greater accuracy. 

### Conclusions

A personalized AI-generated virtual safe place and traditional imagery showed comparable effects, as no significant differences between conditions were observed. These findings support the potential of an AI-generated virtual safe place as a viable alternative for delivering relaxation exercises. The findings offer valuable implications, as this study was the first to investigate and demonstrate the effectiveness of virtual environments in VR personalized with AI.

## Appendix

Multimedia Appendix 1 Exemplary screenshots of the AI-generated safe place.

Multimedia Appendix 2 Correlation matrix of main variables.

Multimedia Appendix 3 Descriptive statistics of RSQ scores, including tests for normality.

Multimedia Appendix 4 Manipulation check.

Multimedia Appendix 5 Sensitivity analysis for main hypotheses H1a and H1b.

Multimedia Appendix 6 CONSORT-EHEALTH (v 1.6.1).

## Abbreviations

IMG: control condition (relaxation while imagining a safe place with closed eyes)
IMG+VR: experimental condition (relaxation in a personalized virtual safe place)
IPQ: Igroup Presence Questionnaire
LDM3D: Latent Diffusion Model for 3D
RSQ: Relaxation State Questionnaire
VR: virtual reality
VVIQ: Vividness of Visual Imagery Questionnaire

## Data Availability

The datasets, including analysis code generated or analyzed during this study, are available from the corresponding author on reasonable request.
