# Supplementary material for: AI-Generated Personalized Visualization of the Safe Place in Virtual Reality vs Traditional Safe Place Imagery: Randomized Controlled Trial
Source: JMIR AI. 2026 Jul 24;5:e82647. doi: 10.2196/82647 (PMC13451872; doi:10.2196/82647)
Supplement: Supplementary file 2 [file ai_v5i1e82647_app1.docx]

Multimedia Appendix 1

Exemplary Screenshots of the AI-generated Safe Place in the Meta Quest 3 VR Headset.

The environments were generated using the following prompts: (A) “Coniferous forest, river, bright summer”, (B) “Sea, sand, beach, sun, very warm”, and (C) “Mountain lake, evening atmosphere, summer weather, forest”.

A)

B)

C)
