# Supplementary material for: AI-Generated Personalized Visualization of the Safe Place in Virtual Reality vs Traditional Safe Place Imagery: Randomized Controlled Trial
Source: JMIR AI. 2026 Jul 24;5:e82647. doi: 10.2196/82647 (PMC13451872; doi:10.2196/82647)
Supplement: Supplementary file 3 [file ai_v5i1e82647_app2.docx]

Multimedia Appendix 2

Correlations Among Key Study Variables

**Table S1**

*Correlations Among Key Study Variables*

| Variable | 1 | 2 | 3 | 4 | 5 | 6 | 7 |
| --- | --- | --- | --- | --- | --- | --- | --- |
| 1. RSQ baseline |  |  |  |  |  |  |  |
| 2. RSQ post-imagery | .52** |  |  |  |  |  |  |
| 3. RSQ post-stress | .60** | .25 |  |  |  |  |  |
| 4. RSQ post-intervention | .56** | .65** | .43** |  |  |  |  |
| 5. RSQ follow-up | .26 | .20 | .09 | .23 |  |  |  |
| 6. IPQ | .23 | .25 | -.07 | .19 | .05 |  |  |
| 7. VVIQ | .36** | .40** | .16 | .50** | .04 | .39* |  |
| 8. Satisfaction | -.08 | -.06 | -.22 | .03 | -.44* | .31 | .07 |

*Note*. Values represent Pearson correlation coefficients (two-tailed). * indicates *P*<.05. ** indicates *P*<.01. Variables assessing presence (IPQ) and satisfaction were only measured in the IMG+VR condition (n = 30). The pairwise sample sizes varied across correlations involving the RSQ follow-up due to missing follow-up data.
