# Supplementary material for: AI-Generated Personalized Visualization of the Safe Place in Virtual Reality vs Traditional Safe Place Imagery: Randomized Controlled Trial
Source: JMIR AI. 2026 Jul 24;5:e82647. doi: 10.2196/82647 (PMC13451872; doi:10.2196/82647)
Supplement: Supplementary file 4 [file ai_v5i1e82647_app3.docx]

Multimedia Appendix 3

Descriptive Statistics of all RSQ Scores Including Tests for Normality

**Table S2**

*Descriptive Statistics of RSQ Scores Including Tests for Normality*

| Time Point | Condition | *M* | *SD* | Shapiro-Wilk *W* | *p* |
| --- | --- | --- | --- | --- | --- |
| RSQ baseline | IMG+VR | 3.07 | 0.56 | 0.97 | .625 |
|  | IMG | 3.21 | 0.65 | 0.96 | .302 |
| RSQ post-imagery | IMG+VR | 3.71 | 0.38 | 0.93 | **.045** |
|  | IMG | 3.77 | 0.49 | 0.97 | .404 |
| RSQ post-stress | IMG+VR | 2.70 | 0.70 | 0.95 | .204 |
|  | IMG | 2.88 | 0.50 | 0.97 | .577 |
| RSQ post-intervention | IMG+VR | 3.51 | 0.47 | 0.90 | **.010** |
|  | IMG | 3.54 | 0.57 | 0.98 | .914 |
| RSQ follow-up | IMG+VR | 3.59 | 0.53 | 0.97 | **.015** |
|  | IMG | 3.42 | 0.56 | 0.99 | .587 |

*Note*. Bold p-values indicate a deviation from normality (p < .050); RSQ = Relaxation State Questionnaire [1]; M = mean; SD = standard deviation; IMG+VR is the experimental condition (intervention is relaxation in a personalized virtual safe place); IMG is the control condition (intervention is relaxation while imagining a safe place with closed eyes).

**References**

1. Steghaus S, Poth CH. Assessing momentary relaxation using the Relaxation State Questionnaire (RSQ). Sci Rep. 2022;12(1):16341. doi:10.1038/s41598-022-20524-w
