# Supplementary material for: AI-Generated Personalized Visualization of the Safe Place in Virtual Reality vs Traditional Safe Place Imagery: Randomized Controlled Trial
Source: JMIR AI. 2026 Jul 24;5:e82647. doi: 10.2196/82647 (PMC13451872; doi:10.2196/82647)
Supplement: Supplementary file 5 [file ai_v5i1e82647_app4.docx]

Multimedia Appendix 4

Manipulation Check

**Table S3**

*Manipulation Check*

| Paired Sample T-Tests | | | | |  |
| --- | --- | --- | --- | --- | --- |
|  | Condition | *t* | *df* | *p* | *Cohen’s d* |
| RSQ baseline – RSQ post-imagery | IMG+VR | -7.17 | 29 | < .001 | -1.31 |
|  | IMG | -5.32 | 29 | < .001 | -0.97 |
| RSQ post-imagery – RSQ post-stress | IMG+VR | 7.23 | 29 | < .001 | 1.32 |
|  | IMG | 9.07 | 29 | < .001 | 1.66 |

*Note.* RSQ = Relaxation State Questionnaire [1]; IMG+VR is the experimental condition (intervention is relaxation in a personalized virtual safe place); IMG is the control condition (intervention is relaxation while imagining a safe place with closed eyes).

**References**

1. Steghaus S, Poth CH. Assessing momentary relaxation using the Relaxation State Questionnaire (RSQ). Sci Rep. 2022;12(1):16341. doi:10.1038/s41598-022-20524-w
