# Supplementary material for: AI-Generated Personalized Visualization of the Safe Place in Virtual Reality vs Traditional Safe Place Imagery: Randomized Controlled Trial
Source: JMIR AI. 2026 Jul 24;5:e82647. doi: 10.2196/82647 (PMC13451872; doi:10.2196/82647)
Supplement: Supplementary file 6 [file ai_v5i1e82647_app5.docx]

Multimedia Appendix 5

Sensitivity Analysis for main Hypotheses H1a & H1b

To evaluate the robustness of the main findings, we repeated both the mixed ANOVA and the linear mixed‑effects model after excluding VR participants who reported low satisfaction with their visualization. Seven participants reported not being satisfied (“Did you receive the picture you wanted?” = “No”) and were excluded from the analyses.

**Mixed ANOVA (sensitivity sample)**

The pattern of results remained unchanged. There was no statistically significant interaction between time of measurement and condition *F*_1,51_=1.83, *P*=.182, *partial η*²=.035. There was a significant main effect for time of measurement *F*_1,51_=82.34, *P*<.001, *partial η*²=.618 and no significant main effect for the condition *F*_1,51_=1.33, *P*=.254, *partial η*²=.025.

**Linear Mixed‑Effects Model (sensitivity sample)**

Using the reduced sample, the linear mixed-effects model yielded the same conclusions as the primary analysis. The main effect of condition remained non‑significant *F*_1, 49.15_=1.41, *P*=.241, indicating that the original results were not driven by low‑satisfaction VR participants.

**Conclusion**

Across both statistical approaches, excluding dissatisfied VR participants did not alter the interpretation of the results.This confirms that the findings are robust and not sensitive to the inclusion of participants who reported very low satisfaction with the VR visualization.
